# Impact of garment construction and environmental factors on heat transfer

**DOI:** 10.1007/s00484-025-02907-6

**Published:** 2025-04-04

**Authors:** Edgar Garcia Torres, Agnes Psikuta, Emiel DenHartog

**Affiliations:** 1 Textile Engineering, Chemistry and Science, North Carolina State University, Raleigh, NC USA; 2 Laboratory for Biomimetic Membranes and Textiles, Empa, Lerchenfeldstrasse 5, St. Gallen, 9014 Switzerland

**Keywords:** Clothing, Insulation, Environment, Heat transfer, Testing

## Abstract

Environmental factors and material construction parameters are both important considerations when evaluating the insulation performance of materials. The environmental factors of air layer thickness, wind speed, and ventilation are known to have a strong impact on insulation in fabric systems. This work builds on this knowledge by examining the interactions between these variables at different levels on various testing apparatuses- the small hot plate, the dynamic hot plate, and the heated torso. Additionally, the work assesses the effect of material air permeability and the influence on insulation at the various environmental conditions. A systematic approach revealed that material air permeability was primarily related to forced convective heat transfer and requires a minimum air layer thickness of 5 mm and wind speed of 1.3 m/s to be observed. Additionally, it was found that traditional methods of evaluating material thickness, such as ASTM D1777, apply a high force to the material and do not give an accurate measurement of uncompressed thickness- which is more indicative of what would be experienced during normal wear conditions. One sample in this study showed a difference > 2x when comparing the ASTM method to an alternative method (KES Compression Test) that applies a lower force, outlining the risk of inaccurate data.

## Introduction

Humans are homeothermic and have different physiological processes to maintain an internal body temperature, or core temperature, of ~ 37 °C (Osilla et al. 2022). This temperature is of the utmost importance and deviations of a few degrees can cause issues such as hyperthermia, hypothermia, and heat exhaustion (Glazer 2005; Osilla et al. 2022). The human body has several physiological processes that can help combat the change in body temperature, such as sweating, shivering, vasodilation and vasoconstriction (Ongwuttiwata and Sudprasertb 2015; Stolwijk and Hardy 1966). Unfortunately, these mechanisms in isolation cannot always compensate for the environmental conditions the user may find themselves in. However, clothing can greatly help the user to regulate their body temperature in different scenarios (Havenith 2002).

Past studies have outlined the importance of material construction when selecting garments with the required insulation for different environments. It was accepted that the thickness of a material was by far the most important parameter when determining the level of insulation required (Gavin 2003; Ukponmwan 1993). However, other studies have shown that the air permeability of fabrics may actually be of similar importance (Havenith et al. 2011; Özkan and Meriç 2015). To the best of our knowledge, there has not yet been a study that controlled the thickness of materials and simultaneously manipulated their air permeability to evaluate the fabric effective insulation. This is reasonable as air permeability is closely linked to the thickness and porosity of the fabric during processing, which makes it difficult to separate the effect of these parameters from one another (Ogulata 2006). Therefore, this study would help understand the effect of fabric air permeability on the resultant insulation of a clothing system.

In addition to the material construction parameters, the environmental and clothing construction parameters also play a critical role in the insulation a clothing system provides. The air entrapped between the clothing and the skin provides additional insulation depending on its thickness. More than 60% of the total insulation of the clothing system can come from this air layer, even up to 80% in some scenarios, and has been found to be paramount in protecting firefighters from burns (Deng et al. 2021; Havenith et al. 1990b; He and Yu 2018; Joshi et al. 2019; Psikuta and Parvus 2023). There exists a critical air layer thickness that can range from 5 to 20 mm that will provide the maximal level of insulation, but this thickness value can vary depending on the application or environment. The effect of air layer thickness on insulation is nonlinear, and factors such as free convection or ventilation greatly impact this relationship and should be defined where possible (Joshi et al. 2019; Song 2007; Špelić et al. 2018). However, the pure insulative power is not the sole effect of the air gap. The air gap will also facilitate heat exchange with the environment through ventilation via the garment fabric and the apertures of the garment (Havenith et al. 1990a; Joshi et al. 2021). Depending on the garment design, this can greatly influence heat exchange with the environment (Joshi et al. 2021). Wind speed is another environmental parameter that can greatly influence the insulation of a clothing system, increasing wind speed is known to lower the total insulation of a clothing system (Holmér et al. 1999; Lu et al. 2015). This phenomenon can be broken down into a few aspects. First, wind will first strip the boundary air layer on the outside of the clothing, the loss of insulation from this layer can be as high as 60% at wind speeds of 1.1 m/s (Nielsen et al. 1985). The wind will penetrate the microclimate via the pores of the fabric and through the apertures of the clothing system, i.e. the sleeves of a shirt, this occurs as wind speed increases and is dependent on the properties of the fabric (Holmér 1988). The overall decrease of insulation via these mechanisms tends to plateau at a wind speed of ~ 3.5 m/s (Burton and Edholm 1955), but this could be observed at wind speeds as low as 2.0 m/s depending on the conditions (Sargolzaei et al. 2021). This study would further build the understanding of these environmental parameters and, crucially, how they interact with each other.

Several methods of evaluating insulation and the effect of these parameters exist and include everything from human trials to relatively simple devices such as the sweating guarded hot plate (Kim et al. 2014). The advantage of the less complex devices is their ease of use, cost effectiveness, and widespread availability. However, the test standards using these apparatuses inconsistently incorporate, or provide guidance, on some environmental parameters, such as microclimate thickness, ventilation, and wind speed (ASTM F1868:2023; ISO 11902:2014). A previous study conducted using a sweating guarded plate was able to measure the effect of air layer thickness and wind but did not incorporate ventilation (Fujimoto et al. 1989). Another study used the sweating guarded hot plate to additionally incorporate the effect of wind speed, air layer thickness, ventilation, and material construction parameters on insulation (Garcia Torres and DenHartog 2024). While the sweating guarded hot plate is a useful apparatus, other testing equipment can provide more realistic conditions. One such device that can be utilized to measure the heat exchange properties of different materials is the sweating heated cylinder Torso (Annaheim et al. 2015; Psikuta et al. 2008). The heated torso is similar to the sweating hot plate in terms of operation principle, but is cylinder shaped to better simulate the trunk of a person and incorporates relevant aspects such as a vertical orientation and crosswind dynamics. Fontana et al. (2017) found the heated torso to give relevant data to assessing the heat exchange mechanism of clothing (Fontana et al. 2017). While the heated torso cannot mimic all unique aspects of the human form, such as distribution of a heterogenous enclosed air layer, the vertical orientation and resultant wind dynamics from the 3 dimensional shape can give more realistic measurements of insulation than the sweating guarded hot plate. Additionally, the thickness of the air gap between the clothing and body can be controlled on the heated cylinder more precisely than on a manikin or person and provide an opportunity to systematically study the effect of specific air gap thicknesses.

The purpose of this study was to evaluate insulation and identify trends that occur at different levels of the environmental parameters such as wind speed, air layer thickness, and ventilation (via fabric construction and apertures in the microclimate). By exploring these parameters in a systematic fashion, the effect of these factors were accurately captured, and crucially, the shift in insulation patterns via the stepwise changes in environment and material construction were able to be observed. This study also analyzed the interactions between these parameters via the factorial effects model, which demonstrated the quantitative relationship between the variables tested. The controlled thickness of the fabrics also allowed the effect of air permeability to be outlined more extensively than in previous studies. The sweating guarded hot plate was used to identify effects of the environment and material construction on a simple device and establish a baseline on a widely available device. Then, these factors were explored on the sweating heated torso to understand the behavior of the parameters on a more geometrically complex device. The findings of this study outline not only the effects of the environment and fabric construction, but critically also the interaction between them. Future studies can introduce sweating to observe the trends of evaporative heat loss as well as incorporate other environmental factors such as solar radiation.

## Methods

### Methodological strategy

A total of 3 measurement devices were used to investigate the effect of the environmental parameters such as wind speed, air layer thickness, and ventilation related to fabric air permeability on heat flux (W/m^2^). Three samples with differing air permeability were used as the outer layer in a double layer configuration. Two sweating guarded hot plate apparatuses were used, as they introduced wind in different directions relative to the fabric surface (parallel and perpendicular). Then, the same materials were tested on the sweating heated torso, which introduced wind across the sample surface. This approach allowed a comparison of heat exchange through a set fabric ensemble on the simplest device - the sweating guarded hot plate - and the data obtained on more complex and realistic geometry of the heated cylinder Torso. This provided an understanding of the behavior of insulation on a simple 2-dimensional device versus a more complex 3-dimensional shape.

### Devices

Two guarded hot plate system and a thermal cylinder were used to evaluate effects of ventilation and air permeability on two-layer fabric systems. The small guarded hot plate at North Carolina State University, NCSU (Raleigh, USA), which is part of the Kawabata Evaluation System- KES (Kawabata et al. 1985) and was located in a climate controlled room. The second apparatus used was the Dynamic Hot Plate (Thermetrics, WA, USA) also located at NCSU which was enclosed in a climatic chamber. The last device used was the heated sweating cylinder called Torso located at Empa (St. Gallen, Switzerland) also enclosed in a climatic chamber. The relevant parameters of each device are listed in Table 1.

**Table 1:** Summary of the parameters of apparatuses used

Device	Small Hot Plate	Dynamic Hot Plate	Heated Torso
Test Area (m^2^)	0.01	0.042	0.43
Wind Direction (relative to sample surface)	Perpendicular	Parallel	Perpendicular
Air gap thickness Used (mm)	0, 5, 10, 25	0, 5, 10, 25	0, 10, 30
Wind Speeds used (m/s)	0.25, 0.5, 1.3	0.5, 1.0	0.5, 1.3, 2.5

**Fig. 1:**
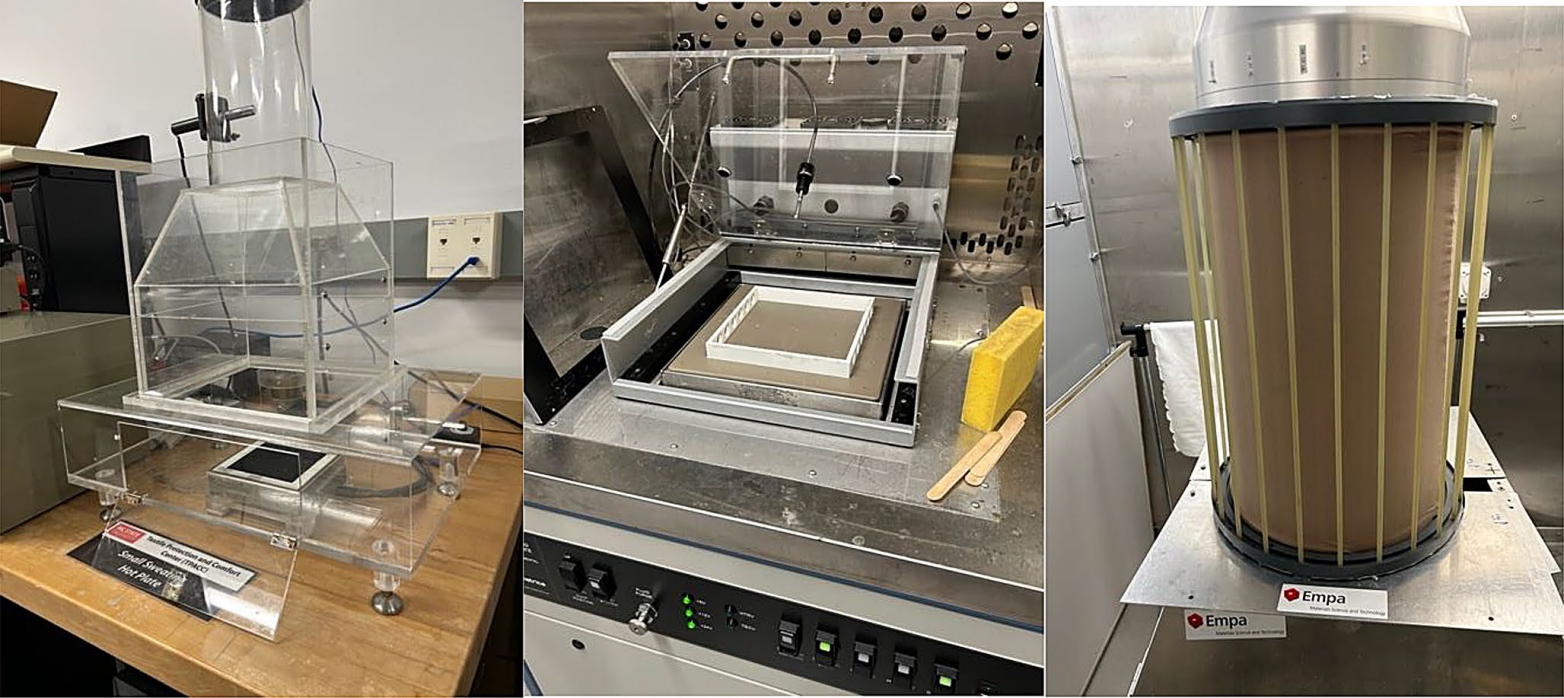
Images of the different devices with the appropriate spacer, small hot plate (left), dynamic plate (middle), and heated torso (right)

Additionally, all devices had an opportunity of introducing additional spacer structures to simulate air gaps. Two levels of ventilation were simulated on both hot plates using different spacer design with and without openings, an example of the devices with a spacer can be seen in Fig. 1. Issues were observed with the dynamic plate in this test set up that made it difficult for the system to reach steady state- elaborated on further in future sections. Note the spacers on both hot plates were the same, except the open design used on the dynamic hot plate only had openings on the two sides not in direct contact with the wind (Fig. 1). This was to avoid the wind blowing through the air gap space. The spacer with the openings allows for more ventilation to occur in the air gap between the two layers of the fabric, while the spacer with no openings inhibits convective heat loss to the environment. The spacers on the heated cylinder were closed on both ends (Fig. 1).

### Fabrics

Three fabric samples of similar thicknesses but with distinct air permeability were chosen to construct the top layer of the 2-layer system. The sample characteristics can be found in Table 2. Base layer sample - the layer closest to the skin, sample A in every test- was placed in direct contact to the apparatus followed by the second fabric layer with or without air gap spacer in between.

**Table 2:** Fabric construction parameters. Note R_ct_ was taken on the dynamic hot plate (Thermetrics) and for a single layer configuration. Values include standard deviation as well

Sample	Material	Structure	Thickness (mm) ASTM D1777	Air Permeability (m^3^/min/m^2^) ASTM D737:2018	*R*_ct_ (m^2^K/W) ASTM F1868:2017
A	100% Cotton	Knit	1.00 ± 0.02	18.9 ± 0.59	0.0802 ± 0.001
B	100% Cotton	Woven	0.68 ± 0.01	5.79 ± 0.34	0.0763 ± 0.0002
C	100% Cotton	Knit	0.64 ± 0.01	54.3 ± 3.4	0.0813 ± 0.0004
D	100% Wool	Knit	0.60 ± 0.01	120. ± 3.3	0.0888 ± 0.001

### Protocols

All tests were conducted at 21 °C of ambient temperature and 65% of relative humidity. All devices were set to maintain constant surface temperature of 35 °C, where the corresponding heating power needed for maintenance of this surface temperature in a given test conditions was assumed to correspond to the heat loss (W/m^2^) through the sample setup. Three repetitions per each combination of sample, air gap and environmental conditions on each device were performed. All combinations of wind speeds and air gap thickness for each device are listed in Table 1, on the hot plates 2 levels of ventilation were also simulated using different spacer design. Before each test samples were acclimatized in environmental test conditions for at least 24 h and the device was preheated and in steady condition. After installing the samples onto the device setup, the measurement was carried out for the period of time ensuring at least 10–20 min of steady state for hot plates and heated cylinder, respectively. The total measurement time approximated 30 min in case of hot plate devices and 60 min in case of heated cylinder. To prevent samples being blown away by the wind a double-sided adhesive tape was used at sample edges to fix it to the guard of the hot plate. However, on the dynamic hot plate this adhesive did not stop the fabrics from “flapping”, or continually shifting, during the test when the spacers were introduced. In case of heated cylinder, the base layer was sewn into a tube shape and slid on to the cylinder. The outer sample was fixed on top using fabric clamps either directly on top of base layer or on the installed air gap spacer.

### Data analysis

The statistical analysis was carried out using JMP PRO 17 software. The parent, or main, effects considered were wind speed, air layer thickness, ventilation, and air permeability. For the analysis, wind speed, air layer thickness, and air permeability were considered continuous variables while ventilation was considered a nominal variable to reflect the “open” and “closed” conditions respectively. The factorial effects model with multiple crossed factors was used to determine the statistical significance and the resultant P-values. When constructing the models, the interaction effects were systematically removed by the number of parameters involved in each layer (i.e., 4th level interactions in the 4th layer involving 4 main effects), then all interactions with a P-value above 0.05 were removed at that layer before moving to the next layer. This process was repeated until all insignificant interaction effects were removed from the model.

## Results

**Fig. 2:**
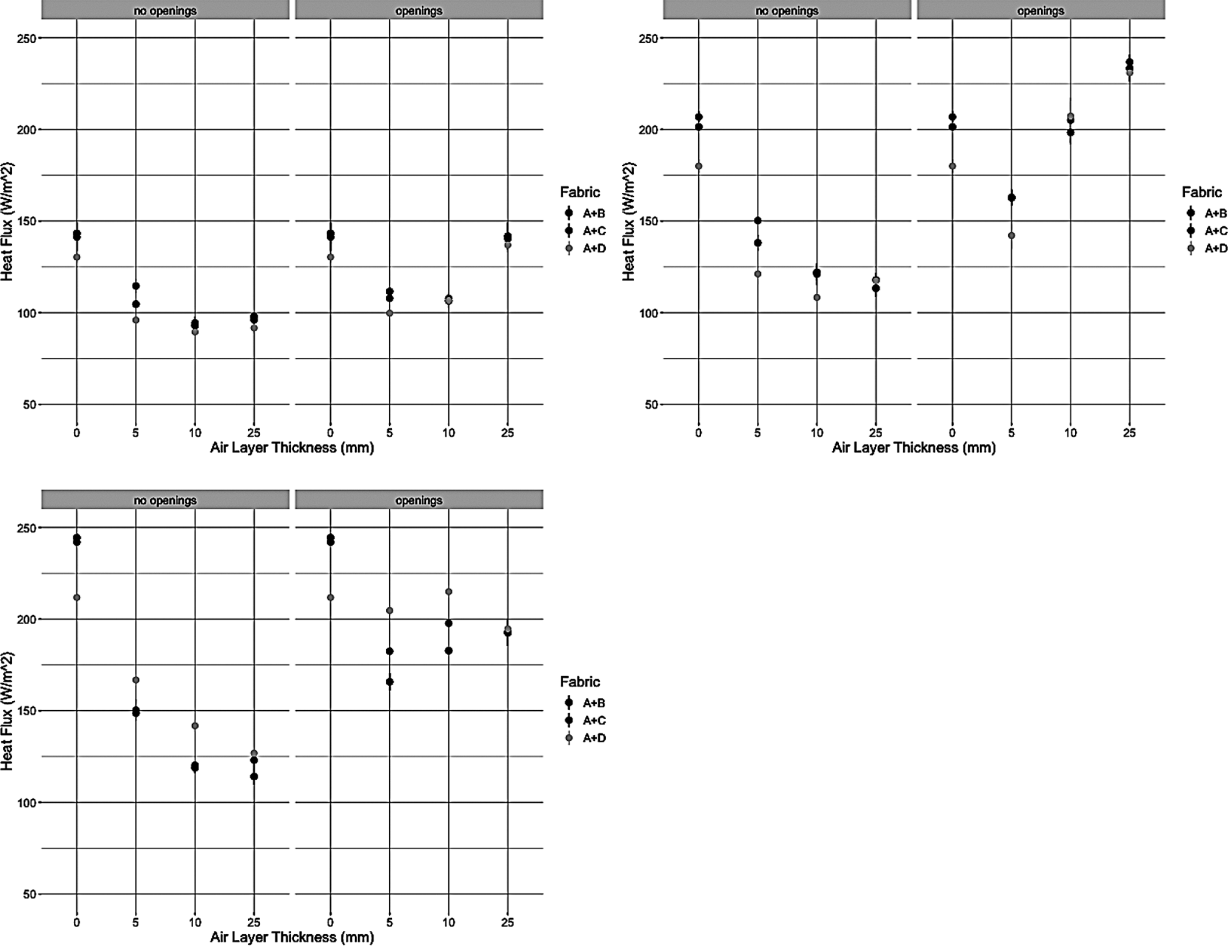
Heat loss results from small plate trials for 0.25 m/s (top left), 0.5 m/s (top right), and 1.3 m/s (bottom left). “No Openings” denotes spacer design without apetures, “Openings” denotes spacer design with apetures

The results of the trials on the small plate are outlined in Fig. 2. The 0.25 m/s wind speed showed relatively small differences between the samples, similar small differences were observed at 0.5 m/s as well. There relative performance in heat flux values of the samples to each other was influenced by the spacer design and air layer thickness combination considered. For example, sample D provided similar heat flux levels to samples B and C at 10 and 25 mm of the air layer thickness with the open spacer design, but was notably lower 0 and 5 mm of air layer thickness. At 1.3 m/s, Sample D gave the highest heat flux levels at all air layer thicknesses except 0 mm on both spacer designs, while sample B yielded the opposite trend.

**Fig. 3:**
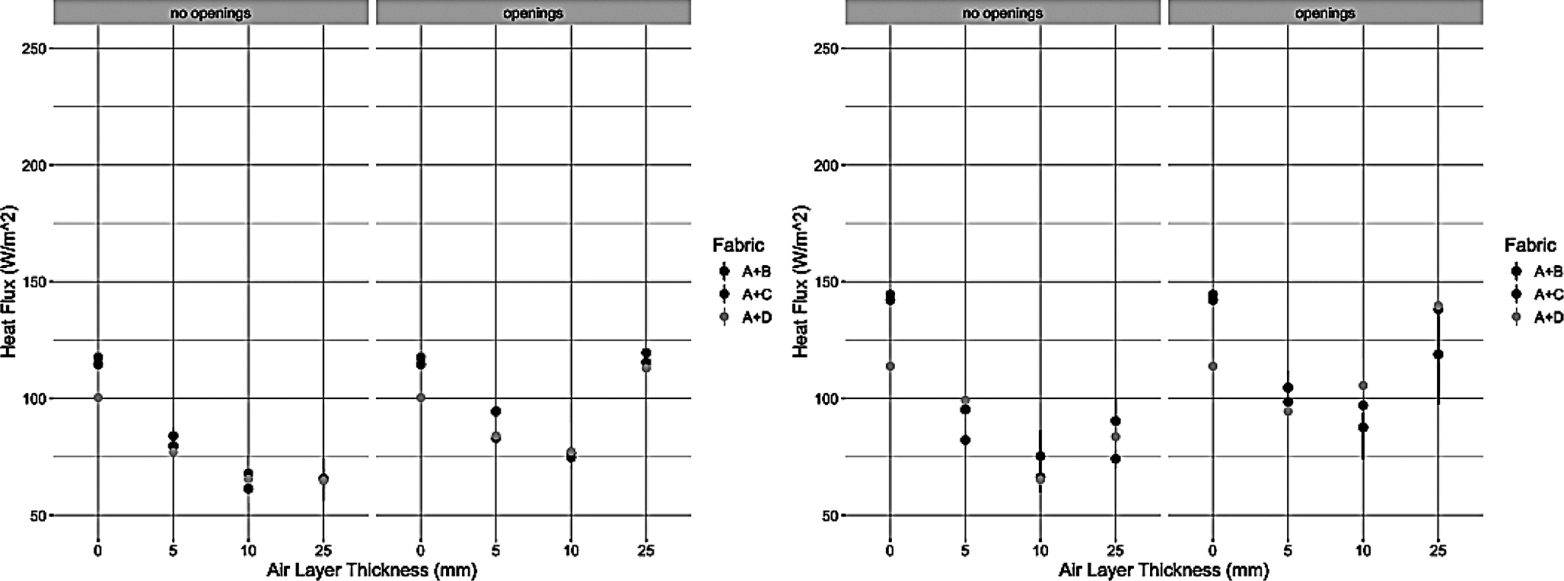
Heat loss results from the dynamic plate at 0.5 m/s (left) and 1.0 m/s (right)

Figure 3 outlines the results of the trials on the dynamic plate. There was notably higher standard deviation than on the small plate trials, especially as wind speed increased. This was due to wind flow causing difficulties for the top layer to stay adhered to the spacer at higher wind speed.

**Fig. 4:**
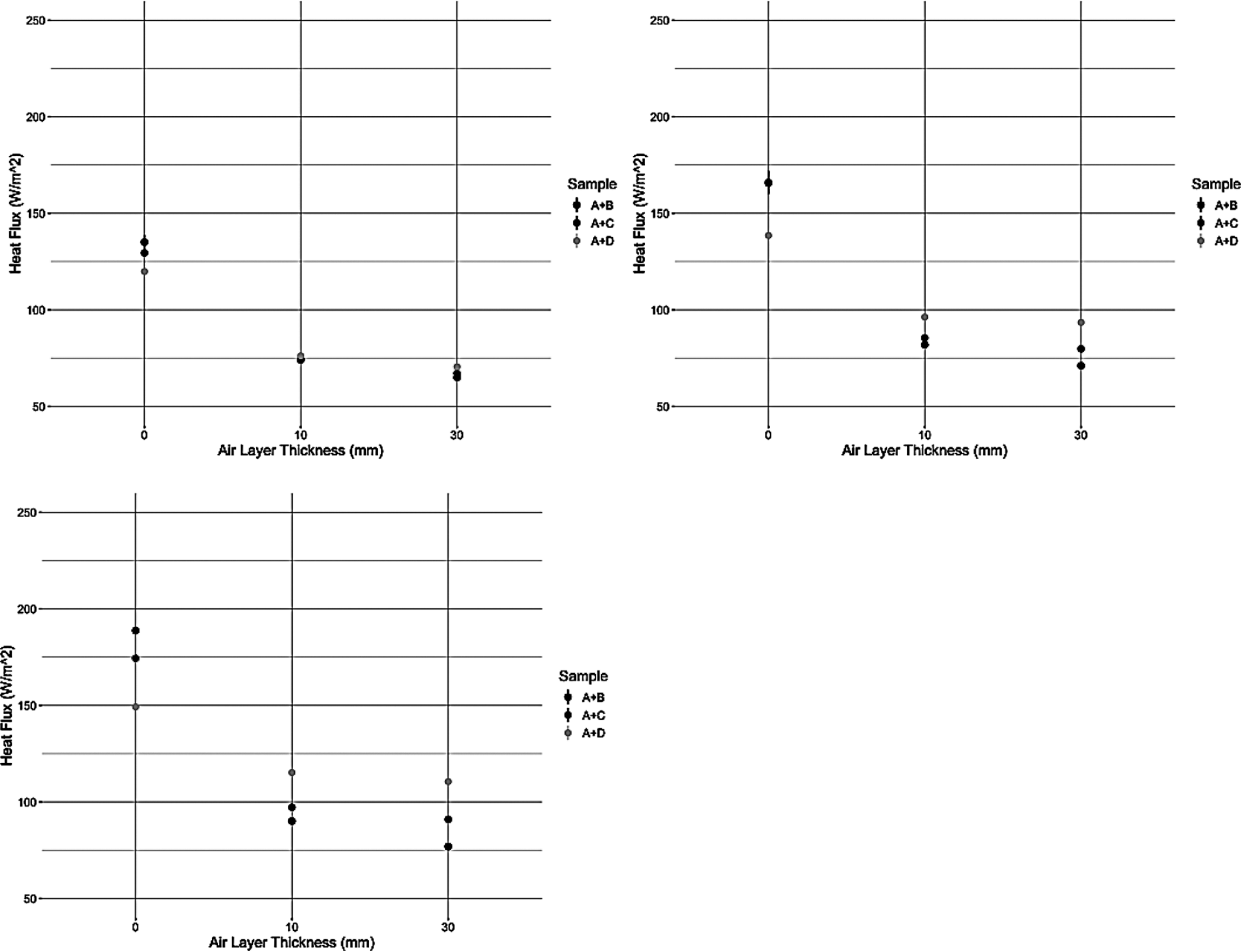
Heat loss results from the heated torso trials at 0.5 m/s (top left), 1.3 m/s (top right), and 2.5 m/s (bottom left). Note the spacer design had no apertures and is classified as “closed”

The results of the torso trials are displayed in Fig. 4. The spacer design of the torso was such that no openings were present in the spacers, this allowed for the material’s construction characteristics to be the sole source of heat exchange with the environment.

Table 3 below demonstrates the statistical results of all the test apparatuses including the main (parent) and interaction effects. Effects were considered statistically significant at *p* = 0.05. Note that a hyphen indicated an effect was not significant while “N/A” meant the effect could not be tested- i.e. on the torso only one spacer design was used therefore ventilation couldn’t be evaluated.

**Table 3:** Statistical results of steady-state insulation values derived from the small hot plate, dynamic plate trails, and torso trials

Effect	Degrees of Freedom	*p*-value (Small plate)	*p*-value (Dynamic plate)	*p*-value (Torso)
Air Layer	1	< 0.0001	0.0037	< 0.0001
Air Permeability	1	-	-	0.6965
Ventilation	1	< 0.0001	< 0.0001	N/A
Air Layer*Ventilation	1	< 0.0001	< 0.0001	N/A
Air Permeability *Air layer	1	-	-	0.0027
Wind Speed	1	< 0.0001	< 0.0001	< 0.0001
Air Layer*Wind Speed	1	< 0.0001	-	-

Table 4 below represents the results of the KES Compression test. Which was used to give an alternative measurement of thickness to ASTM D1777. From the results, it was seen that sample B actually had the lowest thickness, while sample D had the highest thickness. Which was consistent when the single layer was tested and when the samples were tested with sample A as the base layer. This would mean that sample B would be expected to have the lowest insulation, but this was only observed at 0 mm air layer thickness.

**Table 4:** Results of KES compression test, “+A” indicates sample A was used as a base layer in the test

	B	C	D	B + A	C + A	D + A
Compression (%)	46.3	53.3	69.0	33.4	37.2	43.4
Recovery (%)	28.6	24.0	32.4	27.1	24.8	30.8
Thickness (mm)	0.963	1.05	1.37	2.29	2.41	2.58

## Discussion

It can be seen In Figs. 2 and 3 that regardless of the plate apparatuses, the effect of ventilation could be simulated on all the devices. The open spacer design resulted in a decrease of heat flux until 5/10 mm and then would increase at 25 mm air gap thickness, while the closed spacer design showed a decrease of heat flux and then a plateau. These observations became more evident as wind speed was increased, especially on the small hot plate. Increasing of wind speed also led to an increase of heat flux, which is expected as this would increase the convective heat loss thus requiring more power from the test apparatus.

The effect of the top layer was more prominent in the small plate (and Torso) than the dynamic plate. Figure 2 showed that at 0.25 and 0.5 m/s wind speeds there was not a strong effect of the top layer, despite significant differences in air permeability. For example, on the open spacer design Sample D and B both yielded heat flux values of 106 W/m^2^, at 10 mm air gap thickness and 0.25 m/s wind speed, with a standard deviation of ± 4.36 and 7.00 W/m^2^ respectively. Similarly, at 0.5 m/s and 10 mm sample D resulted in 207 ± 14.5 W/m^2^ and B in 198 ± 11.5 W/m^2^. It was determined that the absence of material effect was likely attributed to the low wind speed. At low wind speeds, < 0.5 m/s, only natural convection occurs in the fabric system. These speeds are about what would be experienced in an office setting. In the range of 0.5–1.0 m/s, the air flow is a combination of natural and forced convection in the system, and above 1.0 m/s primarily forced convection is present (Li and Ito 2014; Xu et al. 2021). Therefore, a wind speed of 1.3 m/s was introduced to observe the effect of forced convection. This speed is comparable to modest walking pace (4.7 km/h) and therefore was justifiable to explore. Indeed, in Fig. 2 at 1.3 m/s and 10 m air gap thickness in the open spacer design sample B yielded a heat flux value of 182 ± 1.15 W/m^2^ while D had a value of 214 ± 5.86 W/m^2^. Therefore, it was discerned that to observe the influence of forced convection, an air gap thickness of at least 5 mm and wind speed of 1.3 m/s were required. Then the effect of air permeability of the top layer material was evident. This was observed in both the closed and open space systems. In the open spacer design at 1.3 m/s, sample D showed a higher heat flux of 23% and 17% higher than sample B at 5 and 10 mm. In the closed spacer design, the same was observed but at 11% and 17% higher heat flux respectively. When comparing sample D to C, there was still a difference of 11% and 8% in the open spacer design at 1.3 m/s and 5 and 10 mm respectively. Similarly, in the closed design the difference was 10% and 16% at 5 and 10 mm respectively. The 25 mm spacer design also observed some differences, but they were typically in the 1–5% range, which suggests the air permeability was most influential at air layer thicknesses above 5 mm and below 25 mm. Although the differences were more apparent at 1.3 m/s wind speed, Table 3 showed that air permeability did not yet have a significant effect on heat flux. The wind speed of 1.3 m/s was the highest possible on the small plate. However, all the wind speeds tested had a relevant end use scenario, and these results suggested that top layer air permeability has a smaller effect on heat flux at lower wind speeds.

When examining the statistical results in Table 3, all the environmental variables, wind speed, ventilation, and air layer thickness, were observed to be significant. Additionally, it was seen that there were interaction effects present between the main effects. This was important as it demonstrates quantitatively that these parameters influence each other- not just the final heat flux value. For example, the air layer*ventilation effect showed that the air layer thickness level also influenced the ventilation that was able to occur. I.e. at large at layer thicknesses there is a larger microclimate volume that was able to interact with the mechanism of ventilation. In Fig. 2, this was demonstrated by comparing the trends of the two spacer designs as air layer thickness increased; the closed design demonstrated a plateau while the open design showed a decrease-increase pattern in heat flux values. Similarly, the air layer*wind speed effect indicated that different microclimate volumes interact with the wind speed. This was exemplified by comparing the heat flux values at the same air layers but different wind speeds; an increase of wind speed typically led to an increase of heat flux for all air layers when looking at individual sample trends. Not only could all parameters be observed on the small plate, but the complex interactions be shown analytically. However, on the dynamic plate less effects were observed, and Fig. 3 demonstrated larger variability compared to the small plate. This was likely due the “flapping” of the top layer which could not be addressed despite the use of adhesive tape and thin struts. The dynamic plate cannot be altered to introduce or control wind speed in a different manner and therefore experiments were discontinued on this apparatus due to lack of reliable data.

The results in Fig. 4 on the heated torso showed similar results to the small plate trends with the closed spacer design. When wind speed was above 1.0 m/s, there was more of an effect of the top layer as forced convection was present. At 1.3 m/s wind speed, sample D demonstrated a heat flux value 16% and 27% higher than sample B at 10 and 30 mm air layer thickness respectively. A wind speed of 2.5 m/s resulted in an even greater magnitude of differences when comparing the different surface layers. The heat flux of sample D was 24% and 35% higher than sample B at 10 and 30 mm air layer thicknesses respectively. Table 3 showed a significant effect of Air Layer*Air Permeability (*p* = 0.0027), this meant that an air layer was needed to observe the effect of air permeability. Due to the rule of effect heredity, which states that a parent main effect is always considered as significant when it is present in a statistically significant interaction effect, air permeability was here then considered a significant effect. This was likely due to an air layer allowing for convective heat transfer to occur, without an air layer the primary form of heat loss is through conduction, which appeared to be relatively unaffected by air permeability. The speed of 2.5 m/s is equivocal to 9 km/hr, or 5.6 mph, and comparable to a jogging/running speed. Therefore, it would be expected that as the total wind speed (the user and environment) increased, the effect of the top layer became more apparent. Interestingly, there was also a large difference in insulation at the 30 mm air layer, which would not be predicted from the results of the small plate. This could be a result of the shape of the test apparatus, a flat 2D surface vs. a 3D cylinder, or it could also be that the torso-chamber setup allows for the wind to exit on the rear side with minimal resistance. On the plate as there is a solid surface surrounding the top of the plate, and as such the wind cannot flow in a single direction. It must exit lateral to the plate surface which was not the case for the torso as it was not immediately in front of a wall in the chamber. These considerations may be more prominent at a larger microclimate volume. Regardless, these results demonstrated that there is close relationship between the environmental conditions and material construction parameters.

In all the measurement methods, it was observed that sample B yielded the lowest insulation while D yielded the highest insulation at the 0 mm air layer. This was surprising as sample B was both the thickest and least air impermeable. One traditional method of measuring thickness for insulation, ASTM D1777, utilizes a thickness gauge that imparts a pressure of 250 gf. However, this is an unrealistic force during wear conditions, therefore the Kawabata Evaluation System (KES) Compression test was used to measure thickness at a low pressure (0.5 gf). Which was more representative of what would be experienced during end use. The results from Table 4 showed that sample D was in fact the most thick while sample B was the least thick, at 1.37 and 0.963 mm respectively. Additionally, the compression test measures compression up to 50 gf, and it was seen that sample D was the most compressible, at 69%, and therefore would be subject to larger change in thickness than either of the other samples, 52.3% and 46.3% for sample C and B respectively. These observations remained when the compression test was performed with sample A as the base layer. This explained why at 0 mm air layer thickness sample D yielded the highest insulation. At 0 mm air layer thickness the primary method of heat exchange was via conduction; thus, the most thick sample, D, would indeed yield the highest insulation at 0 mm air layer thickness. Then, as wind speed increased above 1.3 m/s and air layer thickness was at least 5 mm, the air permeability becomes the most influential on the resultant insulation. These are important findings as there often an enclosed air layer present while wearing clothing, and also a total speed (movement + wind speed) of 1.3 m/s is commonly achieved as well. Therefore, the KES compression test results demonstrated that the thickness gauge can give unrealistic measurements of the samples’ uncompressed thickness, which in turn gives misleading ideas on the expectations of the resultant insulation. This is an important consideration in several applications. In human physiology models, fabric thickness is a common parameter entered and therefore inaccurate readings can skew this data. In human subject experiments or product development, materials may be screened based thickness and air permeability. Inaccurate thickness measurements in these cases may result in unnecessary dismal of samples. These are just a few examples that outline the importance of accurate thickness data, sample D in this study showed a difference between the two measurements of a factor greater than 2- which could be even high in thicker materials.

These experiments showed that air permeability has a strong effect on the insulation capabilities of a garment system, but the effect of this property was most influential once the air layer thickness was at least 5 mm and the wind velocity is 1.3 m/s or more. Therefore, when evaluating materials on an apparatus with no air layer present, the effect of air permeability was not so easily observed as it primarily effected forced convective heat transfer. In practical living situations, wind speed and air layer thickness (microclimate) can easily reach the levels tested in these experiments, and therefore there is benefit to the information provided by the modified insulation tests. This was emphasized in Table 3 as the statistical results showed that there was a significant effect of air layer*air permeability. The effect of air permeability was not observed on the small plate due to only 1 wind speed being above 1.0 m/s while 2 wind speeds were tested at 0.5 m/s and below. However, when evaluating materials for insulation, it would be advantageous to introduce an air layer into the testing to observe the effect of convective heat transfer, which is relevant when considering the different fits of garments. Then also to include one wind speed of at least 1.3 m/s, possibly as low as 1.0 m/s, to introduce forced convection into the system. It’s also suggested that the wind flow be perpendicular to the test surface as a parallel configuration could cause similar issues as were observed on the dynamic plate in this study. Unfortunately, no plausible solution was found to reduce the unwanted flapping on the dynamic plate. Models that predict insulation and human response would benefit from this information as it is shown that simulated materials would need to account for the change in insulative properties when considering natural vs. forced convection, when a microclimate volume is present. Additionally, a more realistic measurement of sample thickness using the KES compression test would also improve the qualification of material properties and the expected effect on insulation. Future work would possibility benefit from introducing samples with higher air permeability, but this would be difficult to achieve without altering the thickness of the fabric as well. More wind speeds, especially in the 0.5–1.0 m/s range, would also aid in understanding the transition from natural to forced convection.

## Conclusions

In this work, 2-layer garment systems were tested using several different measurement technologies and it was shown that different levels of the environmental factors wind speed, ventilation, and air layer thickness could be observed on several testing devices. It was also found that air permeability had a significant effect on insulation, however, primarily on forced convective heat exchange. From these trials it was also found that a minimum air layer of 5 mm and a wind speed of at least 1.3 m/s was needed to observe heat loss through forced convection. Therefore, it is recommended insulation testing be conducted on an apparatus that can control microclimate thickness precisely, such as the heat torso. Testing should also include an air layer thickness of 10 mm and wind speeds of 0.5 and 1.3 m/s to observe the effects outlined in this work and be representative of microclimate thicknesses commonly observed during wear (Frackiewicz-Kaczmarek et al. 2015; Psikuta et al. 2018). Additionally, material thickness should be evaluated with an apparatus that can measure at lower forces, such as the KES compression test, to give a more realistic measurement of uncompressed thickness and avoid inaccurate material characterization.

## Data Availability

Data sets generated during the current study are available from the corresponding author on reasonable request.
